# IL-22 hinders antiviral T cell responses and exacerbates ZIKV encephalitis in immunocompetent neonatal mice

**DOI:** 10.1186/s12974-020-01928-9

**Published:** 2020-08-25

**Authors:** Yuejin Liang, Panpan Yi, Wenjuan Ru, Zuliang Jie, Hui Wang, Tamer Ghanayem, Xiaofang Wang, Edrous Alamer, Jinjun Liu, Haitao Hu, Lynn Soong, Jiyang Cai, Jiaren Sun

**Affiliations:** 1 Department of Microbiology and Immunology, University of Texas Medical Branch, 301 University Boulevard, Galveston, TX USA; 2 Department of Infectious Diseases, Key Laboratory of Viral Hepatitis of Hunan, Xiangya Hospital, Central South University, Changsha, Hunan China; 3 Department of Neuroscience, Cell Biology & Anatomy, University of Texas Medical Branch, Galveston, TX USA; 4 Department of Immunology, University of Texas MD Anderson Cancer Center, Houston, TX USA; 5 Department of Pathology, University of Texas Medical Branch, Galveston, TX USA; 6 Department of Medical Laboratories Technology, College of Applied Medical Sciences, Jazan University, Jazan, Saudi Arabia; 7 Institute for Human Infections and Immunity, University of Texas Medical Branch, Galveston, TX USA; 8 Department of Physiology, University of Oklahoma Health Science Center, Oklahoma City, OK USA

**Keywords:** ZIKV, IL-22, Astrocytes, Brain, Microglia, Encephalitis, Neonatal mice, CD8

## Abstract

**Background:**

The Zika virus (ZIKV) outbreak that occurred in multiple countries was linked to increased risk of nervous system injuries and congenital defects. However, host immunity- and immune-mediated pathogenesis in ZIKV infection are not well understood. Interleukin-22 (IL-22) is a crucial cytokine for regulating host immunity in infectious diseases. Whether IL-22 plays, a role in ZIKV infection is unknown.

**Methods:**

The cellular source of IL-22 was identified in *IFNAR*^-/-^ mice and wild-type (WT) neonatal mice during ZIKV infection. To determine the role of IL-22, we challenged 1-day-old WT and *IL-22*^-/-^ mice with ZIKV and monitored clinical manifestations. Glial cell activation in the brain was assessed by confocal imaging. ZIKV-specific CD8^+^ T cell responses in both the spleen and brain were analyzed by flow cytometry. In addition, glial cells were cultured in vitro and infected with ZIKV in the presence of IL-22, followed by the evaluation of cell proliferation, cytokine expression, and viral loads.

**Results:**

We found that γδ T cells were the main source of IL-22 during ZIKV infection in both the spleen and brain. WT mice began to exhibit weight loss, staggered steps, bilateral hind limb paralysis, and weakness at 10 days post-infection (dpi) and ultimately succumbed to infection at 16–19 dpi. IL-22 deficiency lessened weight loss, moderated the systemic inflammatory response, and greatly improved clinical signs of neurological disease and mortality. ZIKV infection also induced the activation of microglia and astrocytes in vitro. Additional analysis demonstrated that the absence of IL-22 resulted in reduced activation of microglia and astrocytes in the cortex. Although IL-22 displayed a negligible effect on glial cells in vitro, *IL-22*^-/-^ mice mounted more vigorous ZIKV-specific CD8^+^ T cell responses, which led to a more effective control of ZIKV in the brain.

**Conclusions:**

Our data revealed a pathogenic role of IL-22 in ZIKV encephalitis.

## Background

Zika virus (ZIKV) is an emerging mosquito-transmitted flavivirus that has caused severe disease in developing fetuses and immunocompromised adults [1–3]. ZIKV infection is a major concern for public health by virtue of its spread to South and Central America in 2014, leading to thousands of human infections in Brazil [4]. Although most adults infected with ZIKV experience a mild influenza-like illness, including fever, headache, rash, conjunctivitis, and joint pain, a minority develops severe nervous system injuries, such as Guillain-Barré syndrome and fatal encephalitis [5]. ZIKV infection also can transmit from the placenta to the fetus in pregnant women and cause gestational abnormalities, including spontaneous abortion, stillbirth, hydrocephaly, and microcephaly [2, 6]. Recent studies demonstrate that several types of brain cells are the targets of ZIKV infection. ZIKV can infect human neural progenitor cells and prevent normal brain growth [7, 8]. Microglia are highly susceptible to ZIKV infection, and they increase inflammatory cytokine production in response [9], indicating the important role of microglia in the pathogenesis of congenital ZIKV infection. Astrocytes are critical for host defense during ZIKV infection, as they are the first cells targeted by ZIKV in the brain [10]. However, ZIKV-infected astrocytes display limited immune responses and may contribute to neuronal infection during later stages by releasing virus [10, 11]. However, key molecules that mediate ZIKV encephalitis are poorly understood.

IL-22 belongs to the IL-10 family and is mainly produced by Th17 cells, Th22, γδ T cells, NKT cells, and innate lymphoid cells [12]. IL-22 primarily targets non-hematopoietic cells, including epithelial, stromal cells, and hepatocytes and promotes cell proliferation and tissue regeneration [13]. IL-22 is reported to play a key role in several inflammatory diseases, such as drug-induced acute hepatitis, inflammatory bowel diseases, pneumonia, asthma, and renal ischemia-reperfusion injury [12]. Accumulating evidence shows that IL-22 contributes to viral infection. We have demonstrated that IL-22 induced atrophy in lymphoid organs, which resulted in poor antiviral T cell responses in the lymphocytic choriomeningitis virus (LCMV) infection [14]. In a lethal West Nile virus (WNV) encephalitis mouse model, IL-22 deficiency resulted in reduced viral load, decreased inflammatory infiltration, and alleviated tissue pathology [15]. These studies suggest that IL-22 may play a detrimental role in the central nervous system (CNS) during viral infection. However, whether IL-22 contributes to ZIKV-induced encephalitis and the underlying mechanisms is not entirely known.

In this study, we found that γδ T cells were the main source of IL-22 in the brain and spleen. We found increased animal survival, improved clinical signs of neurological disease, and reduced viral burden in the *IL-22*^-/-^ neonatal mice. Interestingly, ZIKV infection induced microglia activation and shaped neurotoxic astrocytes [16]. Although IL-22 exhibited a dispensable role for glial cell activation and infection in vitro, the lack of IL-22 resulted in decreased microglia activation in vivo. Importantly, *IL-22*^-/-^ mice mounted more effective ZIKV-specific T cell responses in vivo, while recombinant IL-22 (rIL-22) treatment hindered these responses. Together, our study indicates that IL-22 signaling may play a detrimental role in encephalitis in ZIKV-infected neonatal mice.

## Methods

### Mouse experiments

C57BL/6 (wild-type B6 mice, JAX stock #000664) and *IFNAR*^-/-^ (MMRRC stock #32045) mice were purchased from Jackson Laboratory. *IL-22*^-/-^ mice were kindly provided by Genentech Inc. Mice were housed under 12-h-day/night cycle in the specific pathogen-free, AAALAC-accredited animal facility of UTMB.

ZIKV (Asian lineage FSS13025) was obtained from the World Reference Center for Emerging Viruses and Arboviruses (Galveston, TX). The virus was amplified in Vero cells, and viral titer was calculated as fluorescent focus units (FFU) per milligram. All newborn mice were born from pathogen-free parents and inoculated 1 day after birth with 4 × 10^3^ FFU ZIKV in 2 μL with Hamilton microliter syringes through subcutaneous (*s.c.*) injection at the lateral side of the body. For in vivo treatment, neonatal wild-type (WT) mice were *s.c*. injected with rIL-22 treatment (1 μg in 5 μL, *s.c*.) every other day. In some experiments, mice that were 3 weeks old were intraperitoneally (*i.p.*) infected with 1 × 10^5^ FFU ZIKV. Animals were monitored daily for bodyweight changes and clinical signs of neurological disease. Moribund animals were euthanized in accordance with the UTMB IACUC guidelines.

### Clinical signs of neurological disease

The clinical evaluation of infected neonatal mice was modified according to another report [17]. Briefly, mice were weighed and examined for signs of infection daily. Examination criteria included appearance, stance, and motility. The description of clinical presentations included a staggered step (increased spread of hind legs and unusual pauses during movement), paralysis (loss of muscle function of one or two hind legs), and seizure (sudden stiffening of muscles in the back and legs).

### Cell lines and primary mouse glial cells

Vero and U-87 MG cells were cultured in Minimum Essential Medium (Thermo Fisher Scientific) supplemented with 10% fetal bovine serum (FBS). All cells were cultured in the presence of 100 U/mL penicillin and 100 μg/mL streptomycin in a 37 °C incubator with 5% CO_2_ and 95% humidity control.

For primary mouse glial cell preparation, B6 mouse pups (1–4 days old) were used for mixed cortical cell isolation [18]. Briefly, mouse brains were taken out and placed into a dish containing cold HBSS. The meninges were pulled from the cortex hemispheres with fine forceps under a stereomicroscope to avoid contamination with meningeal cells and fibroblasts. The cortex hemispheres were cut into small pieces, followed by 2.5% trypsin digestion for 30 mins at 37 °C. Cortex tissue pieces were harvested and dissociated into a single-cell suspension. Mixed cortical cells were counted and cultured with DMEM/F12 (1:1) medium plus 10% FBS, 100 U/mL penicillin, and 100 μg/mL streptomycin in a T75 culture flask. The medium was replaced every 5 days. For microglia isolation, the mild trypsinization method was used. Briefly, mixed cortical cells were cultured for 12-15 days and incubated with a trypsin solution (0.25% trypsin, 1 mM EDTA in HBSS) diluted 1:4 in DMEM/F12 medium. The upper layer of cells was detached in one piece and removed from the flask. Microglia remained attached to the bottom and were harvested by further trypsinization for 15–20 min. The purity of microglia was 98.5% as confirmed by CD11b, CX3CR1, and CD45 flow cytometric analysis (Fig. S1A). For astrocyte isolation [19], after 7–8 days in culture, the flask was shaken at 180 rpm for 30 min to remove microglia. The new medium was added into the flask followed by shaking at 240 rpm for 6 hours (hrs) to remove oligodendrocyte precursor cells. Astrocytes were detached by trypsin-EDTA, washed with PBS, and plated into two T75 culture flasks. The medium was changed every 3 days, and astrocytes were harvested at 12–14 days after the first split. Astrocytes were then identified using immunofluorescence (IF) staining of GFAP (Fig. S1B). For in vitro infections, a single dose of ZIKV (MOI of 1) was used.

### RNA isolation and quantitative real-time PCR (qRT-PCR)

RNA was isolated using Rneasy mini kit (Qiagen) according to the manufacturer’s instructions. cDNA was synthesized using the iScript cDNA synthesis kit (Bio-Rad). The abundance of target genes was measured by qRT-PCR using a Bio-Rad CFX96 real-time PCR apparatus as previously described [20, 21]. SYBR Green Master Mix was from Bio-Rad, and TaqMan Universal Master Mix, including gene-specific probes and primers, were from Integrated DNA Technologies (IDT). The amplification efficiency of these primers had been established by means of calibration curves. The total volume for qPCR was 10 μL, comprised of 0.5 μL of each primer (10 μmol/L), 5 μL Master Mix, and 50 ng of cDNA. Nuclease-free water was supplemented to 10 μL. The PCR amplification was as follows: denaturation at 94 °C for 2 min, 40 PCR cycles of 94 °C for 5 s, and 60 °C for 30 s. Finally, a melting step was performed consisting of 10 s at 70 °C and slow heating at a rate of 0.1 °C per second to 95 °C with continuous fluorescence measurement. The relative level of gene expression was calculated using the 2 ^–ΔΔct^ method. The sequences of primers and probes are listed in Supplementary table 1.

### Measurement of viral burden

For measuring viral burden in vivo, mouse tissues were weighed and homogenized using a Tissue-Tearor (BioSpec). ZIKV RNA levels were determined by TaqMan quantitative reverse transcriptase PCR on the real-time PCR detection system (Bio-Rad). The virus burden was determined by interpolation onto an internal standard curve composed of serial 10-fold dilutions of a synthetic ZIKV RNA fragment. A previously published primer set was used to detect ZIKV RNA [22]. For measuring viral burden in infected cells in vitro, the relative level of gene expression was calculated based on C_t_ values using *GAPDH* as a housekeeping gene.

### Lymphocyte isolation and purification

Lymphocytes were isolated according to our previously reported method [23]. Briefly, brains were cut into pieces and digested with 0.05% collagenase IV (Roche, Indianapolis, IN) at 37 °C for 30 min. Cell suspensions were passed through a 70-μm nylon cell strainer to yield single-cell suspensions. Lymphocytes were enriched by centrifugation (400 g) at room temperature for 30 min over a 30/70% discontinuous Percoll gradient (Sigma). The spleens were collected from mice and gently mashed in the RPMI-1640 medium through a cell strainer. Red blood cells were removed by using Red Cell Lysis Buffer (Sigma, St. Louis, MO). Cells were harvested by centrifugation (300 g, 10 min, 4 °C) and resuspended in RPMI-1640 medium plus 10% FBS.

### Flow cytometry

Intracellular staining was performed with flow cytometry as in our previous report [23]. Briefly, for IL-22 and IL-17A detection, lymphocytes were cultured with rIL-23 (20 ng/mL) for 12 hrs. Brefeldin A solution (eBioscience) was added for the last 4 hrs of culture. For detecting IFN-γ and TNF-α in ZIKV-specific CD8 T cells, lymphocytes were incubated with ZIKV peptide E_294–302_ (1 mg/mL, GenScript) in the presence of Brefeldin A solution for 5 hrs. Cells were then stained for anti-CD16/32 (Clone 2.4G2) and surface markers, fixed by using an IC fixation buffer, and followed by staining for intracellular cytokines (Thermo Fisher Scientific). Fixable viability dye, efluor 506 (Thermo Fisher Scientific), was also used to exclude dead cells. All samples were processed on an LSRII FACS Fortessa (Becton Dickinson, San Jose, CA) and analyzed using FlowJo software (TreeStar, Ashland, OR). The flow cytometry antibodies PE-Cy7-conjugated anti-CD3 (17A2), efluor450-conjugated anti-CD4 (GK1.5), APC-eFlour780-conjugated anti-CD8 (53-6.7), FITC-conjugated anti-NK1.1 (OK136), FITC-conjugated anti-TCR gamma/delta (GL3), PerCp-eFlour710-conjugated anti-TNF-α (MP6-XT22), APC-conjugated anti-IFN-γ (XMG1.2), APC-conjugated anti-CD45 (30-F11), Pacific Blue-conjugated anti-CD11b (M1/70), APC-conjugated anti-Ly6G (1A8), FITC-conjugated CD19 (1D3), APC-conjugated anti-IL-17 (eBio17B7), and PE-conjugated anti-IL-22 (1H8PWSR) were purchased from Thermo Fisher Scientific. Purified anti-CD16/32 (2.4G2) and PE-conjugated anti-CX3CR1 (SA011F11) were purchased from Biolegend (San Diego, CA). CFSE dye was used for the cell proliferation assay.

### ELISA

Tissue proteins were extracted using RIPA buffer (Cell Signaling Technology, Danvers, MA) and quantified using a BCA kit (Thermo Fisher Scientific). Mouse IL-22 ELISA kit was purchased from Thermo Fisher Scientific.

### IF staining and confocal microscopy

The IF staining was performed as described previously [24, 25]. Mice were euthanized with CO_2_ and perfused transcardially with cold PBS. Frontal cortices were collected and were immediately placed in 4% PFA in PBS at 4 °C overnight and then cryoprotected in a 30% sucrose solution in PBS for at least 24 hrs at 4 °C. Tissues were embedded in optimal cutting temperature compound (Sakura Finetek, Torrance, CA). Transverse sections (35 μm) were prepared on a cryostat (Leica CM 1900). The sections were kept in Hito floating section storage solution (Hitobiotec Corp) at − 20 °C until they were stained for immunocytochemistry. For immunostaining, tissue sections were rinsed with PBS twice to remove the storage solution and blocked with 5% BSA and 0.3% Triton X-100 in PBS for 2 hrs at room temperature, followed by 48 hrs incubation with primary antibodies. After five washes with PBS, the sections were incubated with fluorophore-conjugated secondary antibodies at 4 °C overnight prior to section mounting. Confocal Z-stacks images were captured within the layer I-II of the cortex using a confocal microscope (Nikon A1). For each mouse, at least 3 fixed-frozen sections were included for each experiment, and at least 3 Z-stacks images at ×20, ×40, or ×60 magnification were taken. Thirty to fifty consecutive optical sections with 1-μm interval thickness at ×40 and ×60 magnification were captured for each Z-stack image. To process images, “Subtract Background” (50 pixels) was applied to remove the background and a threshold (50–225 pixels) was set to remove outliers. The percentages of IBA-1 and GFAP positive areas were analyzed using ImageJ software. Briefly, the positive staining areas were measured first and divided by total areas of the image. For the microglia skeleton analysis, we applied the ImageJ plugin AnalyzeSkeleton and calculated the process length per cell [26]. The rabbit-anti-IBA-1 (#ab178846; 1:500) and goat-anti-GFAP (#ab53554; 1:250) antibodies were purchased from Abcam. The secondary antibodies, including goat anti-rabbit IgG (H + L) Alexa Fluor 488 (#A32731; 1:1,000) and donkey anti-goat IgG (H + L) Alexa Fluor 555 (#A-21432; 1:1,000), were purchased from Thermo Fisher Scientific.

### Western blot analysis

Total brain protein was homogenized in RIPA buffer including a 1% protease inhibitor cocktail (Sigma-Aldrich), and protein concentrations of the lysates were quantified using a BCA kit (Thermo Fisher Scientific). Five to 20 μg of proteins per sample was loaded onto a 12% Novex Tris-Glycine Gel and subsequently transferred to a PVDF membrane. The membrane was blotted with primary antibodies at 4 °C overnight. Antibody detection was accomplished using horseradish peroxidase-conjugated secondary antibodies and visualized with ECL [27]. Markers were used to identify the target protein band. As a loading control, the expression of GAPDH was also measured. The signal intensity was quantified with Image Studio Lite. The primary antibodies anti-VE-cadherin (#ab33168; 1:1000), anti-ZO1 (#ab96587; 1:2000), anti-Occluding (#ab167161; 1:11,000), anti-Claudin 1 (#ab180158; 1:3000), and anti-Claudin 3 (#ab15102; 1:11,000) were purchased from Abcam. Anti-GAPDH (#5174; 1:11,000) was purchased from Cell Signaling Technology.

### Statistical analyses

Data were shown as mean ± SEM and analyzed using the two-tailed Student’s *t* test when compared between two groups. One-way ANOVA was used for statistical analysis of more than two groups. A log-rank (Mantel-Cox) test was used for survival curve analysis. *, **, or *** means *P* value < 0.05, < 0.01, or < 0.001, respectively. Statistical analyses were operated by GraphPad Prism software 7.0 (GraphPad Software Inc., San Diego, CA).

## Results

### ZIKV infection induced γδ T cell-derived IL-22

**Fig. 1:**
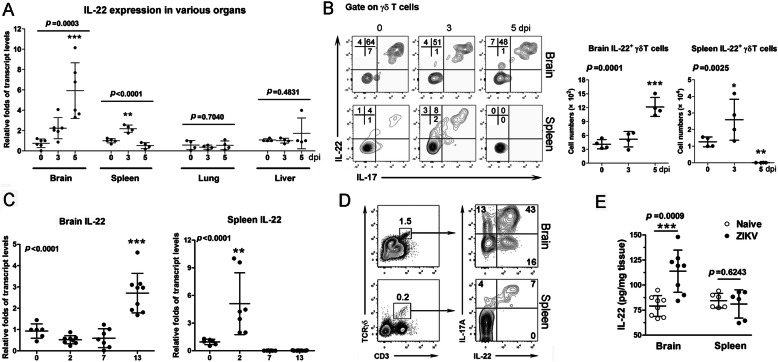
ZIKV infection induces IL-22 expression. **a** Transcript levels of IL-22 were measured in various organs of ZIKV-infected *IFNAR*^-/-^ mice. **b** γδ17 and γδ22 T cells were analyzed in the brain and spleen of ZIKV-infected *IFNAR*^-/-^ mice. **c** IL-22 transcript levels were measured in the brain and spleen of ZIKV-infected neonatal B6 mice. **d** γδ17 and γδ22 T cells were analyzed in the brain and spleen of ZIKV-infected neonatal B6 mice at 13 dpi. **e** IL-22 protein levels were quantified in the brain and spleen at 13 dpi. All experiments were repeated twice independently. A two-tailed Student’s *t* test was used to compare the two groups. One-way ANOVA was used to compare more than two groups. *p < 0.05, **p < 0.01, and ***p < 0.001

To investigate whether ZIKV infection can induce IL-22 expression, we infected 3-week-old *IFNAR*^-/-^ mice with ZIKV as previously reported [28]. We observed that the infected mice started to lose weight at 4 days post-infection (dpi), and all mice died at 6 dpi (Fig. S2A and B). Severe inflammatory infiltration appeared in the liver, lung, and brain at 5 dpi, accompanied with increased expression of inflammatory genes, including *IL-6*, *IL-1β*, *TNF-α*, and *IFN-γ* (Fig. S2C and D). We also found that *IL-22* mRNA expression in the brain increased at 3 dpi and reached a peak at 5 dpi (Fig. 1a), while *IL-22R* mRNA expression displayed a decreasing trend (Fig. S2E). In addition, *IL-22BP* was undetectable in the brain at various time-points (data not shown). Splenic *IL-22* mRNA was upregulated significantly at 3 dpi and then returned to the baseline at 5 dpi. No considerable *IL-22* upregulation was observed in the lung and liver following ZIKV infection (Fig. a). To define the source of IL-22 following infection, we isolated lymphocytes from the brain and spleen and analyzed the IL-22-expressing subpopulations. We found that IL-22 was mainly produced by γδ T cells, which also produced IL-17 (Fig. 1b). NK or conventional T cells had minimal or no detectable levels of IL-22 (Fig. S2F and G). Consistently, the kinetic profile of IL-22^+^ γδ T cells in the brain and spleen showed similar patterns as *IL-22* transcript levels (Fig. 1b). We confirmed this finding using a B6 mouse model by subcutaneously challenging 1-day-old neonatal mice with ZIKV (4 × 10^3^ FFU). The infection of neonates resulted in an elevation of IL-22 expression in the spleen and brain at 2 and 13 dpi, respectively (Fig. 1c). Similarly, we showed that γδ T cells in these immunocompetent mice were the main producers of IL-22 and IL-17 (Fig. 1d). IL-22 protein levels were also increased in the brain, but not in the spleen at 13 dpi (Fig. 1e). Thus, our results indicated that ZIKV infection can induce IL-22 expression by γδ T cells.

### IL-22 deficiency alleviated ZIKV-induced neurological disease

**Fig. 2:**
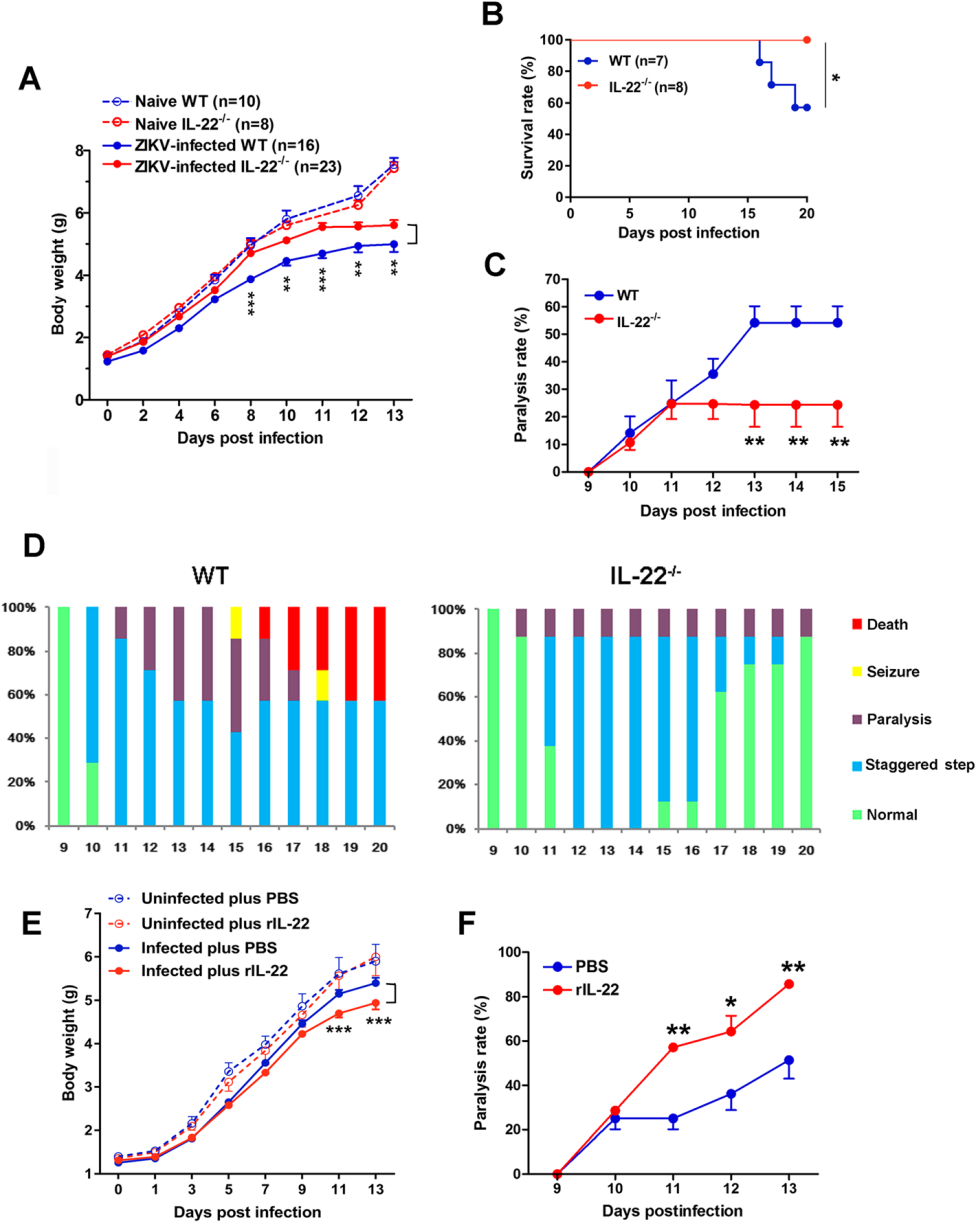
IL-22 deficiency leads to improved clinical signs of neurological disease in ZIKV-infected neonatal mice. Neonatal WT and *IL-22*^-/-^ mice were *s.c.* infected with ZIKV. **a** Body weight, **b** survival rates, **c** paralysis rates, and **d** clinical signs of neurological disease were recorded. **e** Neonatal WT mice were *s.c*. infected with ZIKV, followed by rIL-22 treatment (1 μg in 5 μL, *s.c*.) every other day. Uninfected mice were used as controls. Bodyweights were monitored and statistical analyses were performed between PBS and rIL-22 groups of infected mice (8–10 mice/group) **f** Paralysis rates were generated by pooling the data of three independent experiments (7–9 mice/group). A two-tailed Student’s *t* test was used to compare the two groups. One-way ANOVA was used to compare more than two groups. Log-rank (Mantel-Cox) test was used for survival curve analysis. *p < 0.05, **p < 0.01, and ***p < 0.001

To elucidate the role of IL-22 in ZIKV infection, we *s.c.* infected 1-day-old WT B6 and *IL-22*^-/-^ mice and monitored their bodyweight changes, survival rates, and clinical signs. We found that ZIKV-infected *IL-22*^-/-^ mice maintained significantly higher body weights starting at 8 dpi and continuing to the end of the observation period compared with that of infected WT mice (Fig. 2a). No significant difference in bodyweight was observed between WT and *IL-22*^-/-^ mice without ZIKV infection (Fig. 2a). All *IL-22*^-/-^ mice survived, while about 40% of the WT mice succumbed at 20 dpi (Fig. 2b). Consistently, the rate of paralysis for *IL-22*^-/-^ mice was lower than that of WT mice (Fig. 2c). In the context of clinical signs, WT mice began to display staggered steps at 10 dpi and around 60% of mice developed paralysis or seizure symptoms at 15 dpi. On the contrary, only 10% of *IL-22*^-/-^ mice developed paralysis during the infection (Fig. 2d); neither seizure nor death was observed in the absence of IL-22. Full recovery was observed in about 90% of *IL-22*^-/-^ mice by the end of the study, while none of the WT mice were fully recovered at 20 dpi (Fig. 2d). To further confirm the detrimental role of IL-22, 1-day-old WT mice were infected with ZIKV, followed by rIL-22 treatment every other day. We found that IL-22-treated mice exhibited reduced weight gain at 11 and 13 dpi compared with those of PBS-injected mice (Fig. 2e). Moreover, IL-22 treatment resulted in increased rates of paralysis at corresponding times (Fig. 2f). Collectively, we demonstrated that IL-22 plays a pathogenic role in ZIKV encephalitis.

### IL-22 deficiency reduced microglia activation in ZIKV infection

**Fig. 3:**
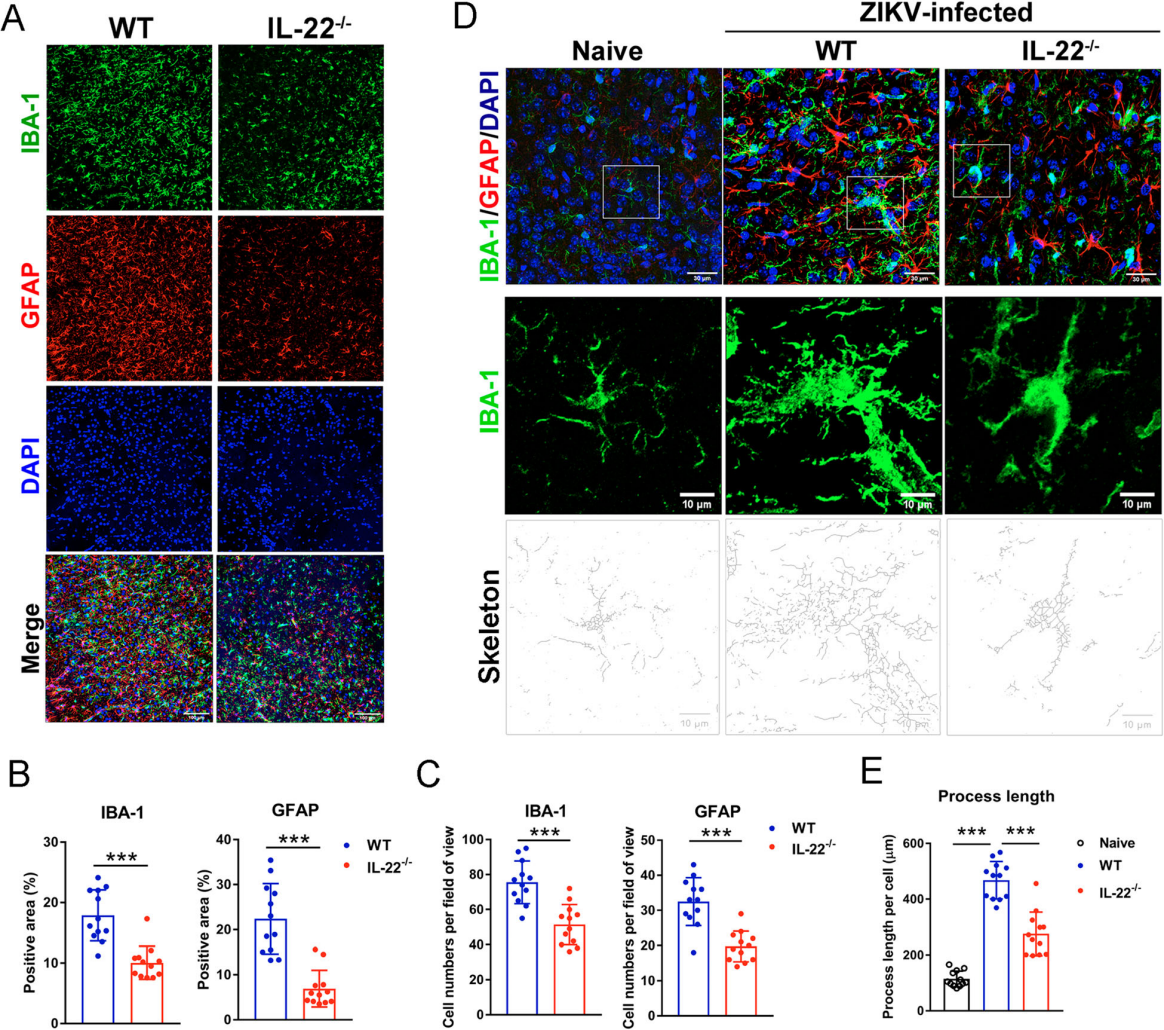
IL-22 deficiency results in decreased microglia and astrocyte activation. Neonatal WT and *IL-22*^-/-^ mice (4–5/group) were *s.c.* infected with ZIKV and sacrificed at 13 dpi. **a** Immunostaining of IBA-1 (microglia cells) and GFAP (astrocytes) in the cerebral cortex. Scale bars, 25 μm. **b** The IBA-1 and GFAP-positive staining areas were measured using ImageJ software. The percentages were calculated as follows: positive staining area/total areas of the image. **c** Cell numbers were expressed as counts/field of view. **d** The morphology of microglia was analyzed using ImageJ Skeletonize2D/3D plugin. The middle row of images represents the rectangular area of view in upper images. The lower images represent the results of skeletonization. **e** The process length was measured using AnalyzeSkeleton plugin of ImageJ and calculated as process length per cell. All experiments were repeated twice independently. Data are shown as means ± SEM. A two-tailed Student’s *t* test was used to compare the two groups in panel **a**. One-way ANOVA was used to compare three groups in panel **e**. ***p < 0.001 and ****p < 0.0001

Microglia cells are considered to be brain residential macrophages that are responsible for the clearance of invading pathogens and damaged neuronal cells [29]. However, hyperactivation of microglia induces chronic inflammation and neurodegeneration [30]. ZIKV-infected microglia exhibit an activated phenotype, characterized by the upregulation of several inflammatory cytokines and may also transmit the virus to other target cells in the brain [9, 31]. To determine the role of IL-22 in the microglial profile during ZIKV infection, we analyzed the microglial phenotype in both WT and *IL-22*^-/-^ neonatal mice. We found that ZIKV-infected *IL-22*^-/-^ neonatal mice showed fewer activated microglia, as evidenced by decreased numbers of IBA-1^+^ cells in the cortex compared to WT control mice (Fig. 3a–c). Similarly, the numbers of activated astrocytes, which were characterized as GFAP^+^, were also lower in the cortex of *IL-22*^-/-^ mice (Fig. 3a–c). Further analysis revealed that ZIKV infection resulted in microglial hyper-ramification; however, the process length of microglia in *IL-22*^-/-^ mice was shorter than those in WT mice, indicating reduced microglia activation in the absence of IL-22 (Fig. 3d, e). We also examined the gene expression of inflammatory cytokines in the brain using qPCR. The mRNA levels of *IFN-γ*, as well as its inducible chemokines, *CXCL9* and *CXCL10*, were not changed significantly. However, the level of brain *TNF-α*, which is the main cytokine produced by microglia [31, 32], was significantly decreased in *IL-22*^-/-^ mice (Fig. S3). Therefore, these data suggested that IL-22-deficiency in ZIKV-infected neonatal mice resulted in reduced microglia activation and decreased pro-inflammatory TNF-α expression.

### IL-22 played a dispensable role in ZIKV-induced glial cell activation in vitro

**Fig. 4:**
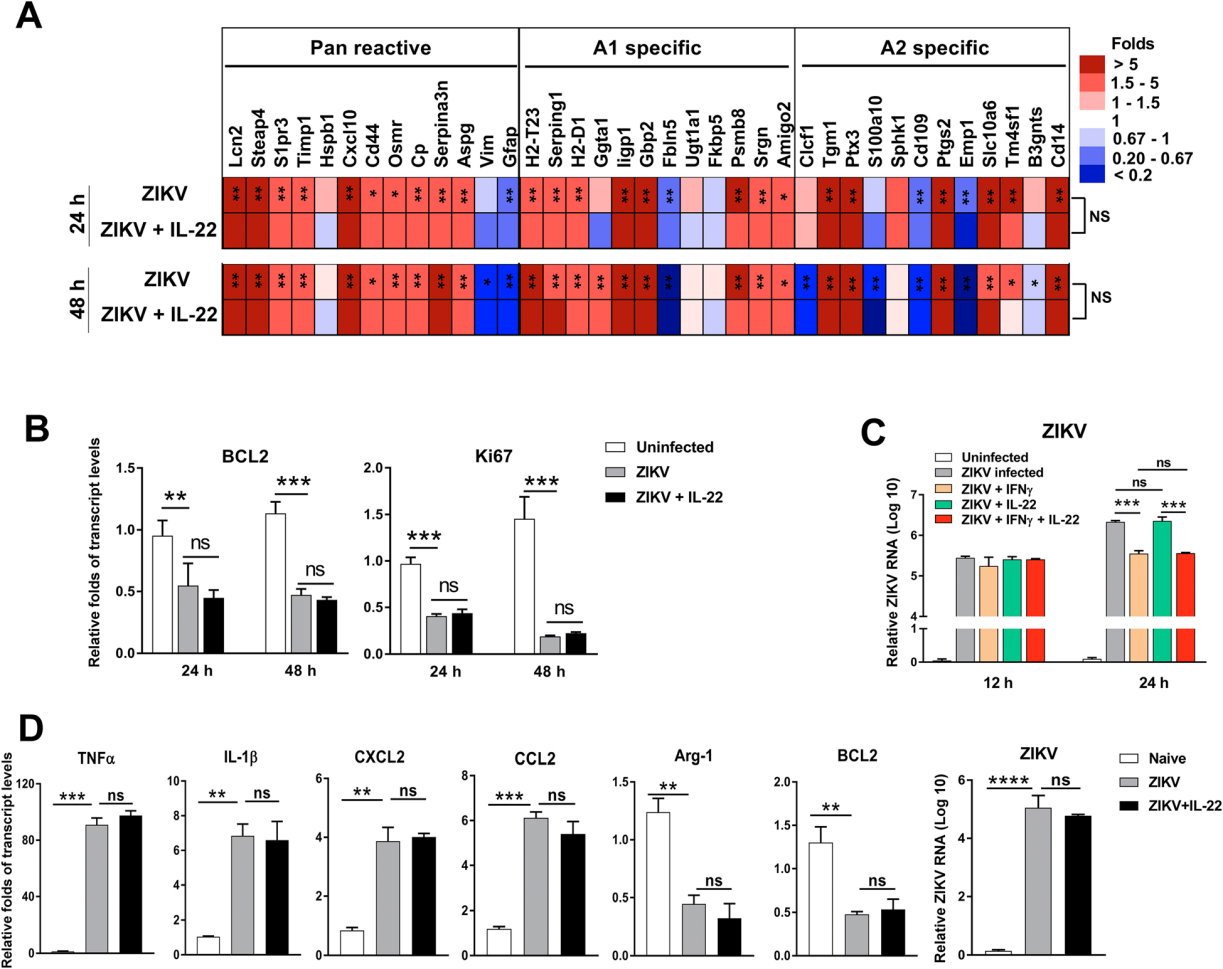
ZIKV induces astrocyte activation, but IL-22 plays a dispensable role in vitro. **a** Mouse primary astrocytes were infected by ZIKV with rIL-22 (200 ng/mL) added or omitted in vitro. Uninfected cells were used as a control. Cells were harvested at 24 and 48 hrs, followed by qRT-PCR analysis for astrocyte activation and (**b**) apoptosis/proliferation markers. In panel **a**, the fold changes of infected groups were normalized to those of uninfected controls. The asterisks in the ZIKV group indicate the results of statistical analysis between ZIKV and control groups. No significant difference was found for any marker between ZIKV and ZIKV+IL-22 groups. **c** Mouse primary astrocytes were infected by ZIKV with or without rIL-22 (200 ng/mL) and IFN-γ (100 ng/mL) in vitro. Viral loads were measured at 12 and 24 hrs. **d** Mouse primary microglia cells were infected by ZIKV with rIL-22 (200 ng/mL) added or omitted in vitro. Transcript levels of inflammatory cytokines and anti-apoptotic marker as well as viral burdens were examined by qPCR. All experiments were repeated twice independently. Data are shown as means ± SEM. Each group contains at least three samples, and one-way ANOVA was used to compare three groups. *p < 0.05, **p < 0.01, ***p < 0.001, ****p < 0.0001, NS, not significant

To determine whether IL-22 has a direct effect on glial cell activation and viral inoculation, mixed cortical cells were isolated from mouse pups and cultured in vitro for generating astrocytes and microglia (Fig. S1). Reactive astrocytes mainly display two polarizations, termed A1 and A2, which play neurotoxic and protective roles, respectively [16]. We found that ZIKV infection induced astrocyte activation as a mixed A1/A2 phenotype. However, IL-22 did not show any effect on ZIKV-induced astrocyte activation (Fig. 4a). To determine whether IL-22 contributes to cell apoptosis and proliferation, we analyzed *BCL2* and *Ki67* transcript levels in ZIKV-infected astrocytes. Although ZIKV infection resulted in decreased cell survival and proliferation as evidenced by the downregulation of *BCL2* and *Ki67* expression, no effect of IL-22 was observed on astrocytes in vitro (Fig. 4b). Finally, we measured viral loads in ZIKV-infected astrocytes in the presence of IL-22. Our data showed that ZIKV-infected astrocytes efficiently and IFN-γ significantly decreased viral burden at 24 hrs. However, neither IL-22 alone nor synergized with IFN-γ altered viral burdens (Fig. 4c). To further confirm our results, we infected human glial cell U-87MG with ZIKV, followed by rIL-22 treatment. Our results showed that IL-22 did not rescue cell death or promote cell proliferation (Fig. S4 A and B). The comparable transcript levels of *CXCL10*, *CCL2*, and *BCL2*, as well as viral loads were observed between control and rIL-22-treated groups (Fig. S4 C and D), indicating that IL-22 was dispensable for the growth, activation, and viral infection of human glial cells. Microglia, as resident macrophages in the brain, have a high ability to secret inflammatory cytokines (e.g., TNF-α), which induce neurotoxic astrocytes and lead to neuron death [16]. Our qPCR data showed that ZIKV-infected mouse primary microglia increased the expression of several inflammatory cytokine/chemokine genes, including *TNF-α*, *IL-1β*, *CXCL2*, and *CCL2* (Fig. 4d). Arginase-1 (Arg-1) can compete with nitric oxide synthase in the brain, playing a neuroprotective role [33]. We found reduced *Arg-1* expression in microglia following ZIKV infection, suggesting to us that ZIKV may cause damage in the brain through inhibiting neuroprotective factors. In addition, ZIKV-infected microglia downregulated anti-apoptotic gene *BCL2* expression, indicating that ZIKV infection may promote glial cell apoptosis (Fig. 4d). Although microglia were infected by ZIKV, IL-22 did not contribute to this infection or cell activation, as evidenced by similar levels of viral loads and comparable gene expression of activation markers following IL-22 supplementation in vitro (Fig. 4d). To elucidate the reason why glial cells failed to respond to IL-22, we measured *IL-22R* transcript levels and revealed no detectable level of *IL-22R* in both microglia and astrocytes (data not shown). We also found no overt changes in examined tight junction and adherence junction proteins, including ZO-1, VE-cadherin, Occludin, Claudin-1, and Claudin-3 in the brain of both ZIKV-infected WT and *IL-22*^-/-^ mice (Fig. S5). Therefore, our data demonstrated that ZIKV infection induced activation of astrocytes and microglia, leading to brain inflammation, while IL-22 was dispensable for glial cell activation and viral clearance in vitro.

### IL-22 hindered anti-ZIKV CD8^+^ T cell responses

**Fig. 5:**
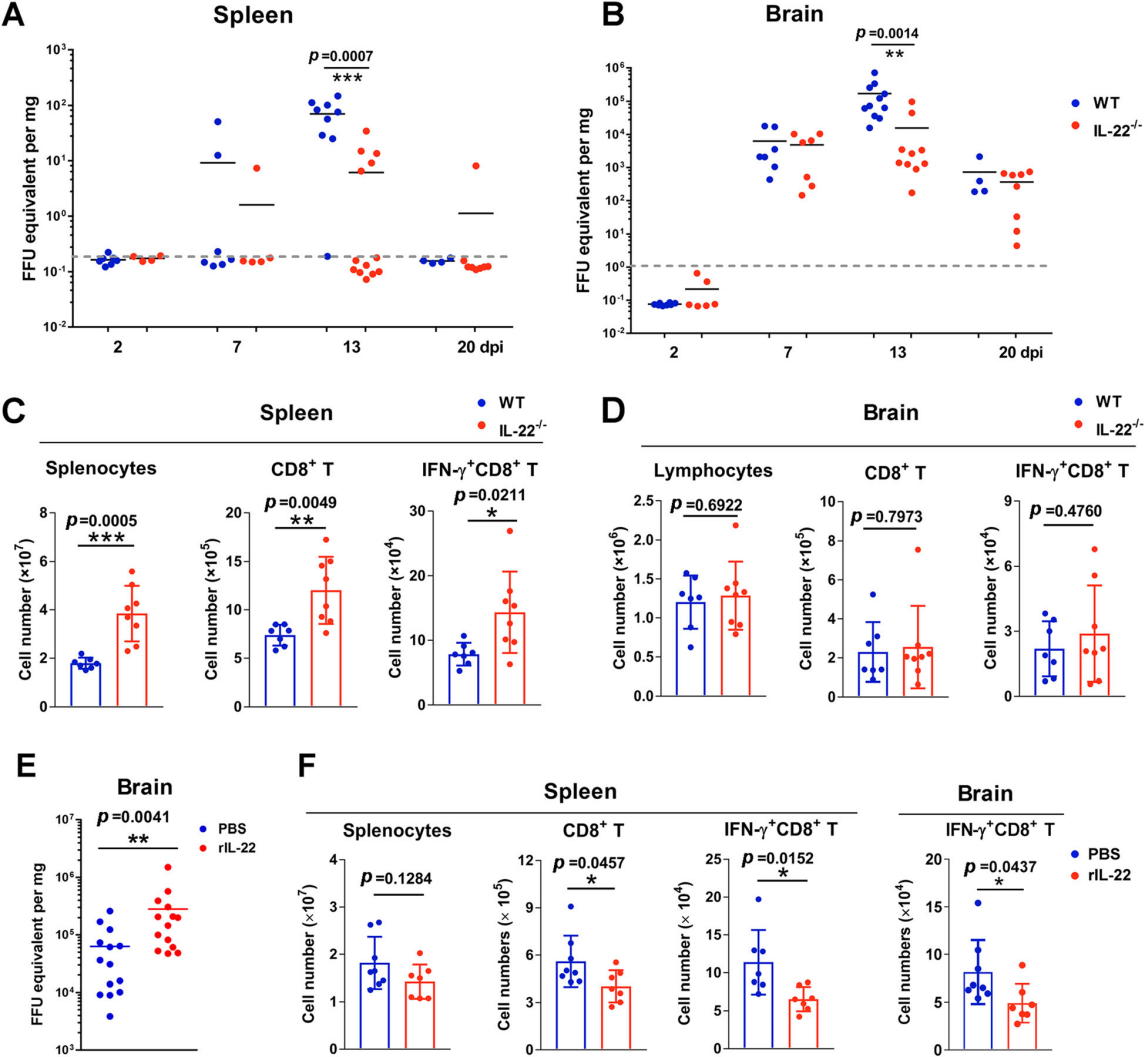
IL-22 dampens anti-ZIKV CD8^+^ T cell responses. Neonatal WT and *IL-22*^-/-^ mice were *s.c.* infected with ZIKV. **a**, **b** Viral loads of the spleen and brain were measured at 2, 7, 13, and 20 dpi. **c**, **d** Lymphocytes were harvested from the spleen and brain at 13 dpi and stimulated with ZIKV peptide for 5 hrs in the presence of Brefeldin A. ZIKV-specific CD8^+^ T cells were quantified by intracellular flow cytometry staining. **e** Neonatal WT mice were *s.c.* infected with ZIKV, followed by rIL-22 treatment as indicated in Fig. 2**e**. Viral loads of the brains were measured at 13 dpi, and **f** ZIKV-specific CD8^+^ T cells were quantified in the spleen and brains. All experiments were repeated three times independently. Data are shown as means ± SEM and a two-tailed Student’s *t* test was used for statistical analysis. *p < 0.05, **p < 0.01, and ***p < 0.001

Although immune cells do not express IL-22 receptor, IL-22 can regulate T cell responses in both viral and parasitic infections, probably via indirect ways [14]. The adaptive immune response, especially the anti-ZIKV cytotoxic CD8 T cell response, has been demonstrated to play a protective role against ZIKV infection [34, 35]. However, excessive CD8^+^ T cell infiltration in the brain can cause paralysis in mice with ZIKV infection [36]. Here, we speculated that the absence of IL-22 resulted in more robust CD8^+^ T cell responses, which efficiently controlled ZIKV replication and dissemination. To test this hypothesis, we *s.c.* infected 1-day-old WT and *IL-22*^-/-^ mice and investigated viral burden and anti-ZIKV CD8^+^ T cell responses. We found that tissue viral load spiked at 13 dpi in WT mice, with much higher levels in the brain compared with those in the spleen (Fig. 5a, b). Importantly, *IL-22*^-/-^ mice displayed significantly lower viral loads in both the spleen and brain at the peak of viral infection (13 dpi), but not at other time-points including 2, 7, and 20 dpi (Fig. 5a, b). We further analyzed anti-ZIKV CD8^+^ T cells and found the comparable numbers infiltrated IFN-γ^+^CD8^+^ T cells in the brains of WT and *IL-22*^-/-^ mice at both 10 and 13 dpi. However, *IL-22*^-/-^ mice displayed more effective anti-ZIKV CD8^+^ T cell responses in the spleen at 13 dpi, as evidenced by increased numbers of IFN-γ^+^CD8^+^ T cells (Fig. 5c, d and S6A). These results may indicate that IL-22 deficiency promoted effector functions of antiviral CD8^+^ T cells without increasing inflammatory infiltration to the brain. Consistently, exogenous IL-22 treatment increased brain viral loads and impaired anti-ZIKV CD8^+^ T cell responses in both the spleen and brain (Fig. 5e, f). We also confirmed our finding using a 3-week-old immunocompetent mouse model. Again, IL-22-deficiency resulted in increased cytokine-producing CD8^+^ T cells in the spleen at 7 dpi (Fig. S6B). Collectively, our findings suggested that IL-22 dampens anti-ZIKV T cell responses in the periphery and exacerbates viral infection in the brain, leading to profound cerebral inflammation and animal paralysis and death.

## Discussion

The roles of IL-22 in various diseases are diverse. In mucosal disorders, IL-22 plays a protective role by preserving epithelial integrity [37, 38], promoting antibacterial peptides and proteins [39], and inducing mucins [40, 41]. However, IL-22 is pathogenic in some inflammatory settings, such as psoriasis [42], allergic airway inflammation [43], and collagen-induced arthritis [44]. The role of IL-22 in viral infection remains enigmatic. Our previous research indicated that IL-22 contributes to antiviral immune responses and determines viral clearance in LCMV infection [14]. In this study with ZIKV infection, we found that IL-22 deficiency resulted in decreased viral loads, alleviated clinical manifestations, and increased survival rates in neonatal mice (Fig. 1, 3, and 3). Our in vitro results showed that ZIKV infection promoted the activation of astrocytes and microglia, leading to neuroinflammation in the brain [16]. Although IL-22 did not directly exert its effects on brain glial cells in vitro, the absence of IL-22 led to reduced glial cell activation in vivo (Fig. 4). More importantly, *IL-22*^-/-^ mice generated qualitatively better ZIKV-specific T cell responses compared with those in WT mice. Therefore, our results demonstrated a pathogenic role of IL-22 in ZIKV encephalitis of neonatal mice.

Upon viral infection, IL-22 induction is organ-specific. NK and NKT cells can produce IL-22 in response to murine cytomegalovirus and influenza virus infection [45–47]. Intrahepatic γδ T cells are also the source of IL-22 in hepatitis B virus-infected patients [48]. We have previously reported that intrahepatic γδ T cells are the main immune cells to produce IL-22 by IL-23 stimulation in an LCMV-infected mouse model [14]. Similarly, we found in this study that γδ T cells were the main source of IL-22 in the spleen and brain in both *IFNAR*^-/-^ and WT neonatal mouse models (Fig. 1). The peak of IL-22 expression in the spleen was as early as 2 dpi, while the time-point for peak expression of IL-22 in the brain was delayed. Since ZIKV initially infected lymphoid organs and subsequently invaded the CNS [28], this dynamic pattern of IL-22 suggests that IL-22 might be driven by the virus or virus-induced innate immune responses. Indeed, ZIKV infection induced high levels of brain inflammatory cytokines, including *IL-1β* (Fig. S1D), which may facilitate the expression of IL-22 from γδ T cells [49]. In addition, high levels of *IL-6* in the brain, but not in the liver and lung, also suggest that IL-6 may be required for IL-22 production [50].

Both WNV and ZIKV belong to the Flaviviridae family and cause severe encephalitis. It has been reported that *IL-22*^-/-^ mice were resistant to lethal WNV infection due to reduced inflammatory infiltration and decreased viral load in the CNS [15]. Infection of both WNV and ZIKV in *IL-22*^-/-^ mice led to alleviated clinical manifestations (Fig. 2) with decreased viral load and elevated pro-inflammatory *TNF-α* expression in the brain (Fig. 5a and S3) [15], whereas rIL-22 cytokine treatment in vivo played a detrimental role in ZIKV infection (Fig. 2e). Interestingly, there were several distinct aspects between WNV and ZIKV infection. First, IL-22 was critical for virus-carrying neutrophil migration through the blood-brain barrier, leading to severe WNV infection in the brain [15]; however, ZIKV-infected *IL-22*^-/-^ mice showed similar levels of lymphocyte infiltration, chemokine expression, and junction proteins in the brain (Fig. 5d, S3 and S5). It is reported that neutrophil migration from the blood into the brain was strikingly reduced in WNV-infected *IL-22*^-/-^ mice [15]. Although we observed neutrophil infiltration in the brain during ZIKV infection, the absence of IL-22 did not change the number of infiltrated neutrophils (data not shown). Secondly, IL-22 deficiency did not contribute to the anti-WNV immunity in the periphery [15], but actually resulted in more vigorous ZIKV-specific CD8^+^ T cell responses in the spleen (Figs. 5c and S6). In line with recent findings that CD8^+^ T cells protected against ZIKV infection in the CNS [35, 51, 52], our finding suggests that IL-22 may contribute to ZIKV encephalitis pathogenesis via modulating periphery CD8^+^ T cell responses. Additional evidence is that IL-22 deficiency did not influence WNV burdens in the spleen [15]; however, *IL-22*^-/-^ mice had lower viral loads in the spleen following ZIKV infection (Fig. 5a). The possible reasons for these discrepancies could be related to the diversity of the two viruses and differences of the animal models.

ZIKV can target several types of glial cells, including astrocytes and microglia, leading to intracranial viral spreading, brain inflammation, and fetal congenital malformations [2, 9, 10]. Microglia-derived TNF-α plays a critical role as an inflammatory mediator and can further activate microglia through an autocrine manner [32, 53]. Neutralization of TNF-α or depletion of microglia prevents memory impairment in ZIKV-infected mice [54], indicating that microglia and TNF-α play detrimental roles in ZIKV infection. We showed in this study that ZIKV infection caused hyper-ramification of microglia (Fig. 3) [29]. We also found that IL-22 deficiency resulted in reduced microglia numbers as well as cell activation (Fig. 3a, b). In addition, decreased *TNF-α* expression was observed in the brain of *IL-22*^-/-^ mice (Fig. S3). Therefore, our in vivo results suggested that IL-22 may contribute to glial cell activation and induce brain inflammation, leading to cerebral pathogenesis. In addition to microglia, astrocytes are considered the initial target of ZIKV infection immediately following viral inoculation of newborn mice [10]. A recent study explored whether activated microglia can produce inflammatory factors (e.g., TNF-α, IL-1β*,* and complement C1q) to induce astrocyte polarization into neurotoxic A1 astrocytes, which secrete neurotoxins and rapidly degenerate neurons and mature differentiated oligodendrocytes [16]. Our in vitro data comprehensively demonstrated a pan-reactive as well as a mixed A1/A2 phenotype of astrocytes during ZIKV infection (Fig. 4a). Our data indicated that ZIKV not only infects astrocytes for viral replication, but also directly induces astrocyte activation. Moreover, ZIKV-activated microglia may also facilitate astrocyte activation through the production of TNF-α and IL-1β, which were highly expressed in the brain following ZIKV infection (Fig. S1D).

Whether IL-22 can directly affect glial cells and regulate cell function is not entirely clear. IL-22 receptor was detected in human astrocytes of both healthy controls and multiple sclerosis patients, and IL-22 treatment reduced TNF-α-induced apoptosis of astrocytes [55]. In addition, exogenous IL-22 also promoted the proliferation of human glial cells accompanied by an anti-apoptotic effect [56]. To our surprise, such effects of IL-22 on astrocytes or microglia were not observed in our study using a human glial cell line, mouse primary astrocytes or microglia (Figs. 4 and S4). Although ZIKV infection significantly inhibited cell growth and elevated inflammatory gene expression, supplementing with IL-22 was dispensable for cell proliferation and activation. Moreover, neither IL-22 alone nor synergizing with IFN-γ contributed to ZIKV replication in astrocytes and microglia (Fig. 4c). In addition, we were unable to detect *IL-22R* transcript in mouse primary astrocytes and microglia (data not shown). These data suggest that IL-22 may not be capable of directly regulating glial cell function in the brain; instead, IL-22 appears to dampen antiviral T cell responses and delay viral clearance in the periphery, leading to increased ZIKV invasion, glial cell activation, and disease severity. However, we cannot exclude the possibility that IL-22R may be inducible and upregulated by inflammatory cytokines in the brain due to the disease status. Additionally, IL-22 may need a synergetic mechanism with critical cytokines, such as IFN-λ, to amplify downstream signals and execute its function [57]. Although the reason for the discrepancies in ours and others’ studies is not known at present, further investigation is needed to clarify the unique role of IL-22 in the CNS among distinct disease animal models.

## Conclusions

Taken together, our studies demonstrated that ZIKV infection promoted microglia and astrocyte activation, whereas the absence of IL-22 resulted in reduced glial cell activation and improved clinical signs of disease in a neonatal mouse model of ZIKV encephalitis. Mechanistically, IL-22 dampened anti-ZIKV T cell responses and delayed viral clearance, leading to exacerbation of glial cell activation and brain inflammation. In all, our study suggests that the neutralization of IL-22 may be a potential therapeutic against ZIKV encephalitis.

## Supplementary information

**Additional file 1.** Supplementary data.

**Additional file 2: Table S1.** Primer pairs for qRT-PCR assays.

## Data Availability

All data generated or analyzed during this study are included in this published article and its supplementary information files.

## Abbreviations

ZIKV: Zika virus
WT: Wild-type
dpi: Days post-infection
LCMV: Lymphocytic choriomeningitis virus
WNV: West Nile virus
CNS: Central nervous system
FFU: Fluorescent focus units
*s.c.*: Subcutaneous
hours: hrs
