# Supplementary material for: IL-22 hinders antiviral T cell responses and exacerbates ZIKV encephalitis in immunocompetent neonatal mice
Source: J Neuroinflammation. 2020 Aug 25;17:249. doi: 10.1186/s12974-020-01928-9 (PMC7448338; doi:10.1186/s12974-020-01928-9)
Supplement: Supplementary file 1 — Additional file 1. Supplementary data. [file 12974_2020_1928_MOESM1_ESM.docx]

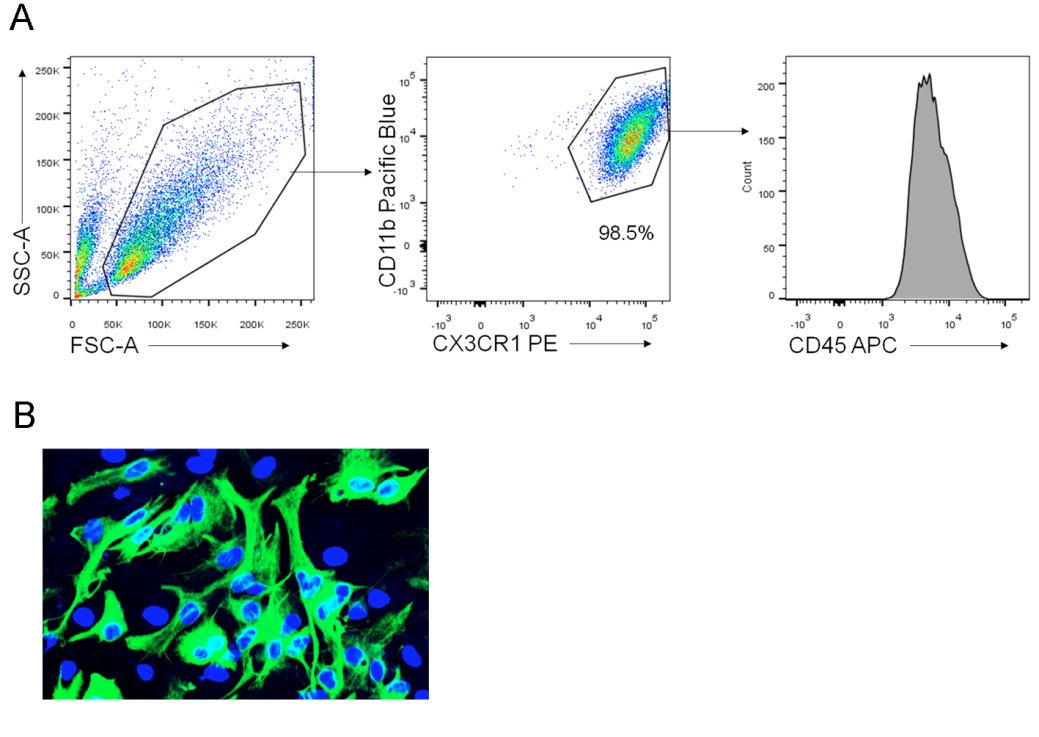


**Figure S1. Identification of mouse primary microglia and astrocytes.** (A) Mouse microglia were stained with CD45, CD11b and CX3CR1 fluorescent antibodies and analyzed by flow cytometry. (B) Immunofluorescence staining of astrocytes using GFAP (green) and DAPI (blue).


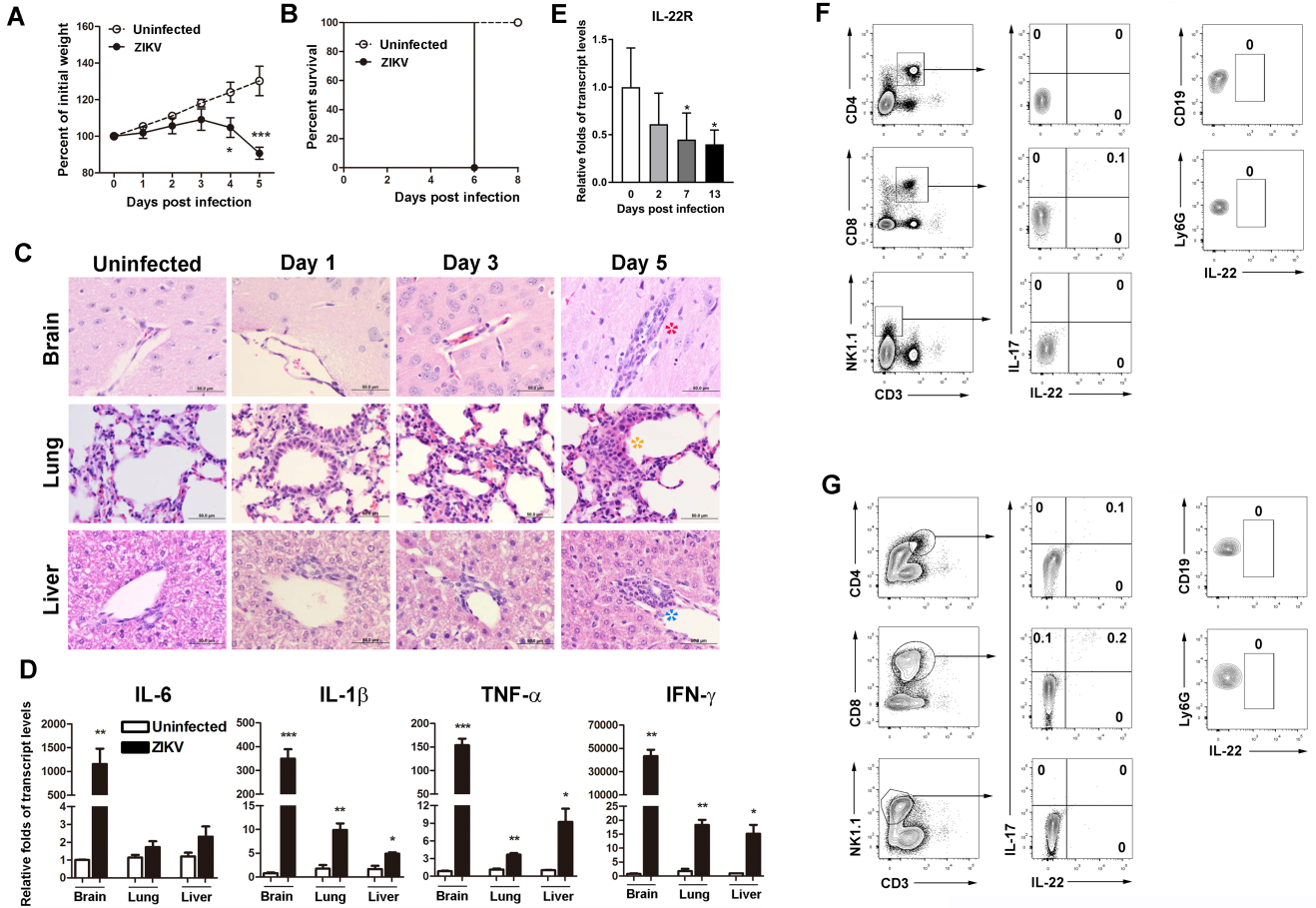


**Figure S2. ZIKV infection causes systemic inflammation in IFNAR^-/-^ mice.** IFNAR^-/-^ mice (5-6/group) were infected with ZIKV (1×10^5^ FFU/mouse, *i.p.*). (A) Bodyweight changes. (B) Survival rate. (C) Histological changes in the brain, lung, and liver tissues. Perivascular cuffing or encephalitis in the brain (red asterisk); Peribronchial inflammation in the lung (yellow asterisk); and periportal inflammation in the liver (blue asterisk). (D) The transcript levels of IL-6, IL-1β, TNF-α, and IFN-γ were detected by qRT-PCR in the brain, lung and liver tissues at 5 dpi. (E) Neonatal WT mice were *s.c.* infected with ZIKV. Brain tissues were collected at 0, 2, 7 and 13 dpi, followed by qRT-PCR analysis for *IL-22R*. Lymphocytes were isolated from (F) spleen and (G) brain at 13 dpi for IL-22 expression analysis by flow cytometry. All experiments were repeated twice independently. Data are shown as means ± SEM and a two-tailed Student’s t-test was used for statistical analysis. * *p*<0.05, ** *p*<0.01, *** *p*<0.001.


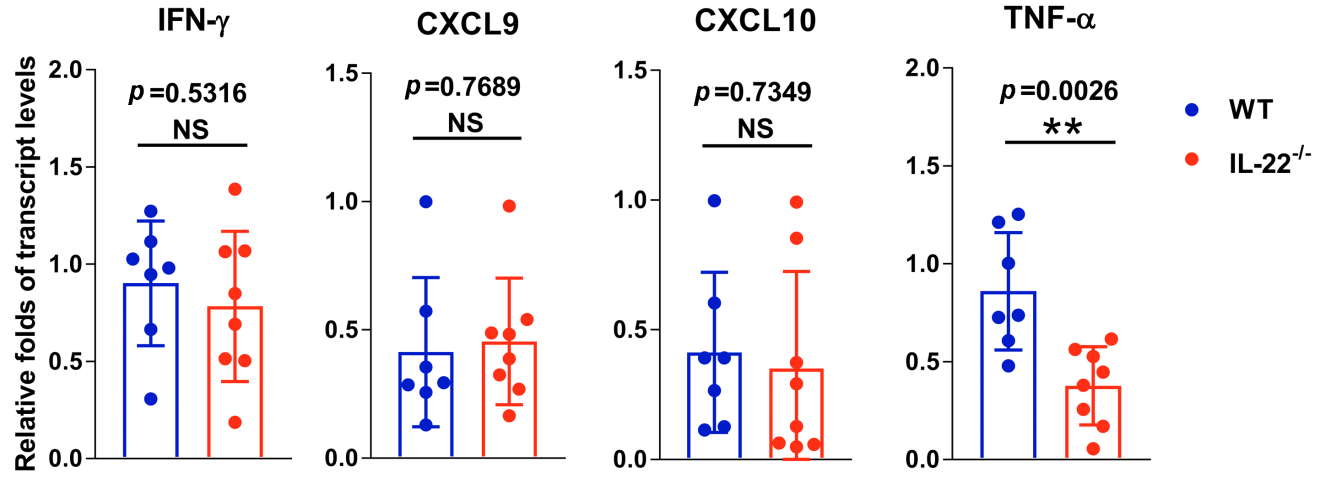


**Figure S3. IL-22 deficiency results in decreased TNF-α expression in brains.** Neonatal WT and *IL-22*^-/-^ mice were *s.c.* infected with ZIKV. Brain tissues were collected at 13 dpi, followed by qRT-PCR analysis for inflammatory genes. This experiment was repeated twice independently. Data are shown as means ± SEM and a two-tailed Student’s t-test was used for statistical analysis. ** *p*<0.01, NS, not significant.


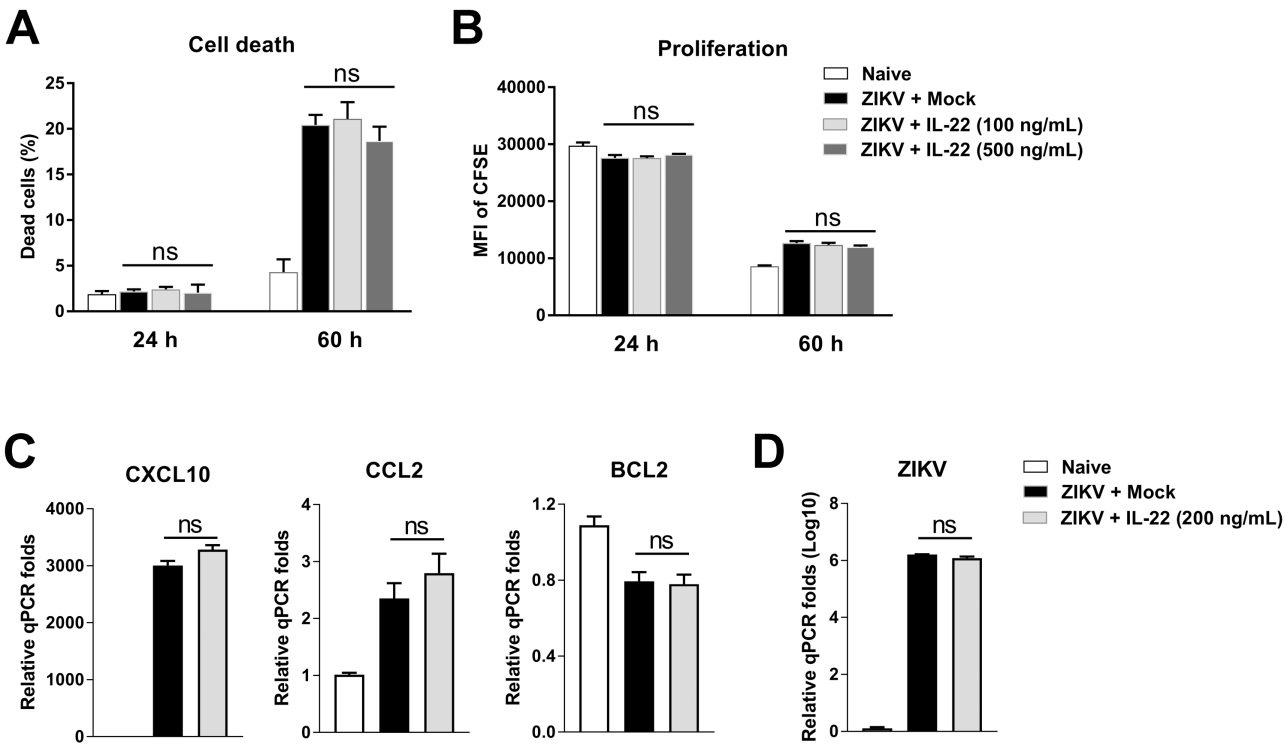


**Figure S4. IL-22 plays a negligible role in human glial cell proliferation and activation following ZIKV infection.** Human glial cell line (U87mg) was infected by ZIKV (MOI 1) with or without rIL-22. (A) Cells were harvested at 24 and 60 hrs, stained with fixable live/dead cell dye and analyzed by flow cytometry. (B) Cells were labeled with CFSE before ZIKV infection and harvested at 24 and 60 hrs for cell proliferation assay. (C and D) Transcript levels of CXCL10, CCL2 and BCL2, as well as ZILV viral loads, were analyzed by qRT-PCR at 24 hrs of infection. These experiments were repeated three times independently. Data are shown as means ± SEM and one-way ANOVA was used to compare multi-groups for statistical analysis. NS, not significant


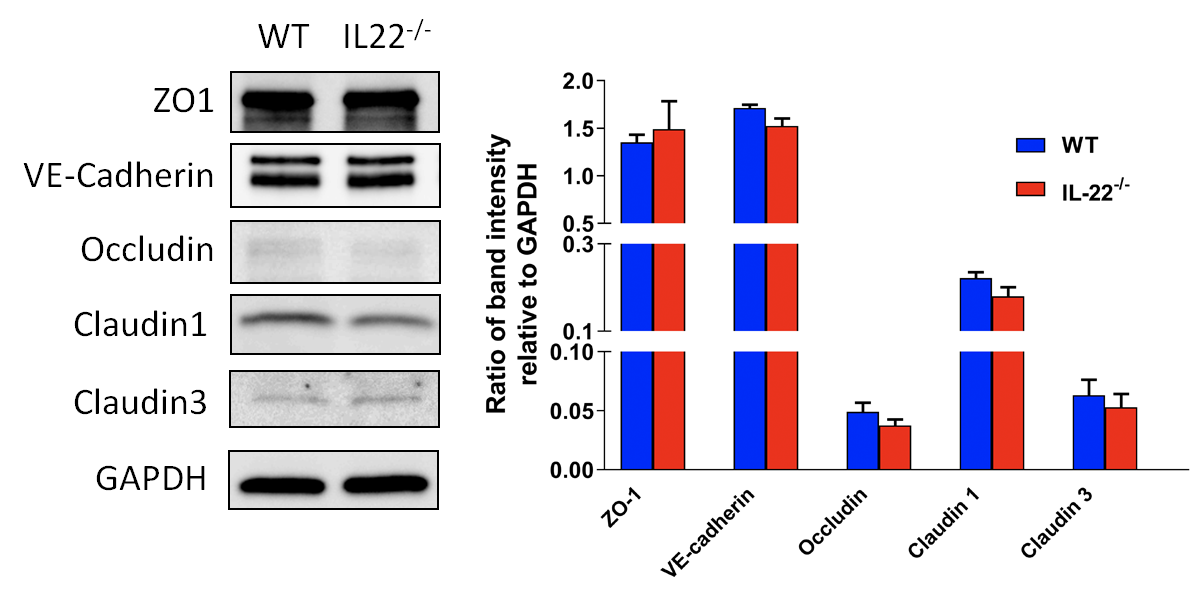


**Figure S5. Junction proteins were not altered in brains of *IL-22^-/-^* mice following ZIKV infection.** Neonatal WT and *IL-22*^-/-^ mice were *s.c.* infected with ZIKV. Brain tissues were collected at 13 dpi and analyzed for tight junction proteins by western blot assay. Each group contains 4-5 mouse brain samples. Data are shown as means ± SEM and a two-tailed Student’s t-test was used for statistical analysis. Immunoblot bands were quantified using Image Studio Lite Ver 5.2 and the intensity was determined and normalized to a respective loading control protein GAPDH.


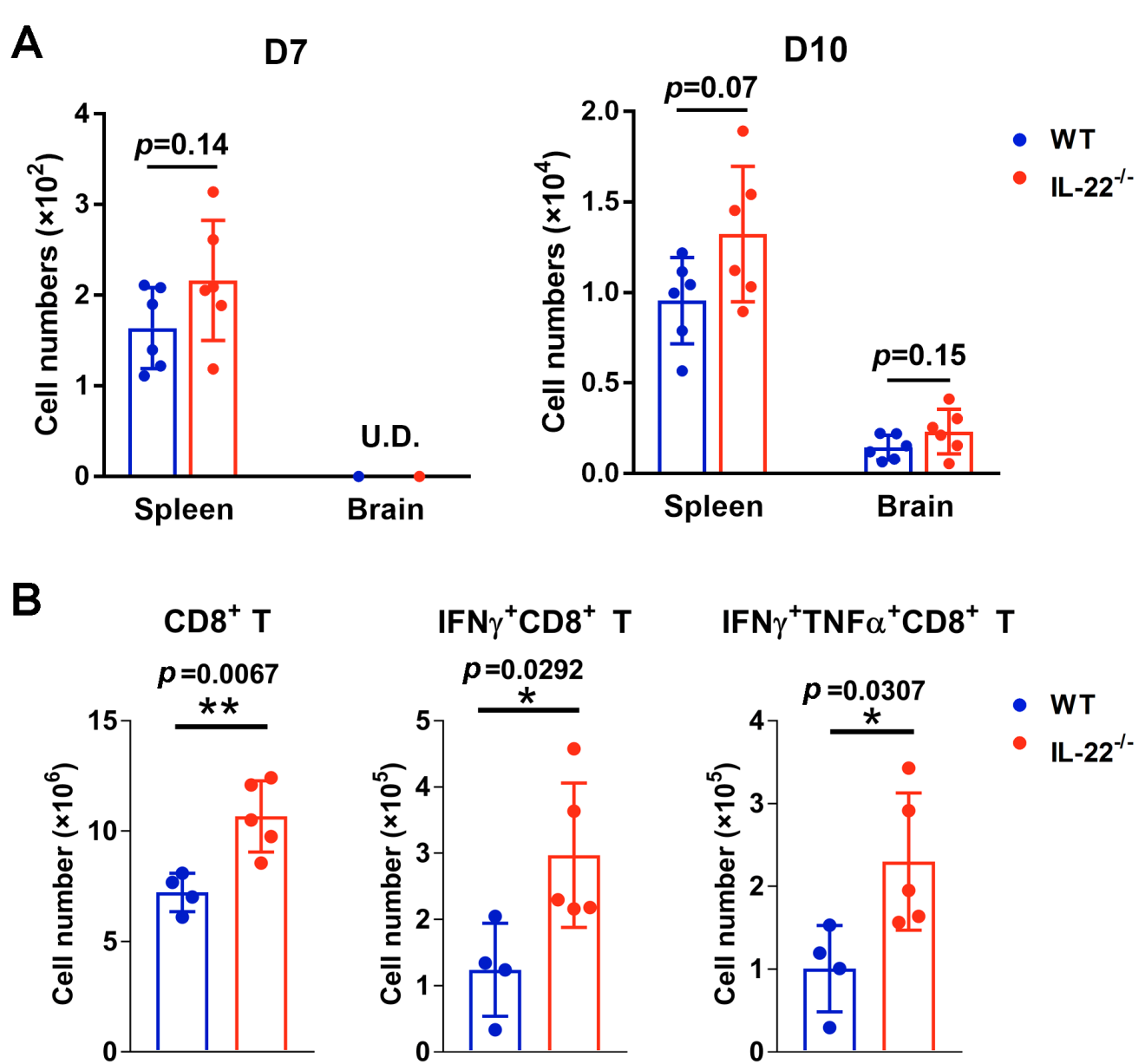


**Figure S6. *IL-22^-/-^* mice display stronger anti-ZIKV CD8^+^ T cell responses.** (A) Neonatal WT and *IL-22*^-/-^ mice were *s.c.* infected with ZIKV and sacrificed at 7 and 10 dpi (B) WT and *IL-22*^-/-^ three-week-old mice were *i.p.* infected with ZIKV and sacrificed at 7 dpi. Lymphocytes were stimulated with ZIKV peptide for 5 hrs in the presence of Brefeldin A. ZIKV-specific CD8^+^ T cell responses were quantified by intracellular flow cytometry staining. This experiment was repeated twice independently. Data are shown as means ± SEM and a two-tailed Student’s t-test was used for statistical analysis. * *p*<0.05, ** *p*<0.01.
