# Supplementary material for: IL-22 hinders antiviral T cell responses and exacerbates ZIKV encephalitis in immunocompetent neonatal mice
Source: J Neuroinflammation. 2020 Aug 25;17:249. doi: 10.1186/s12974-020-01928-9 (PMC7448338; doi:10.1186/s12974-020-01928-9)
Supplement: Supplementary file 2 — Additional file 2: Table S1. Primer pairs for qRT-PCR assays. [file 12974_2020_1928_MOESM2_ESM.docx]

**Table S1. Primer pairs for qRT-PCR assays**

**Gene Name Primer Sequence**

**Mouse:**

GAPDH Forward 5’-TGGAAAGCTGTGGCGTGAT-3’

Reverse 5’-TGCTTCACCACCTTCTTGAT-3’

IFN-γ Forward 5’-ATGAACGCTACACACTGCATC-3’

Reverse 5’-CCATCCTTTTGCCAGTTCCTC-3’

TNF-α Forward 5’-CCCTCACACTCAGATCATCTTCT-3’

Reverse 5’-CTTTGAGATCCATGCCGTTG-3’

Arg-1 Forward 5’-CTCCAAGCCAAAGTCCTTAGAG-3’

Reverse 5’-AGGAGCTGTCATTAGGGACATC-3’

CXCL2 Forward 5’- CCAACCACCAGGCTACAG-3’

Reverse 5’- GCGTCACACTCAAGCTCTG-3’

Lcn2 Forward 5’-CCAGTTCGCCATGGTATTTT-3’

Reverse 5’-CACACTCACCACCCATTCAG-3’

Steap4 Forward 5’-CCCGAATCGTGTCTTTCCTA-3’

Reverse 5’- GGCCTGAGTAATGGTTGCAT-3’

S1pr3 Forward 5’-AAGCCTAGCGGGAGAGAAAC-3’

Reverse 5’-TCAGGGAACAATTGGGAGAG-3’

Timp1 Forward 5’-AGTGATTTCCCCGCCAACTC-3’

Reverse 5’-GGGGCCATCATGGTATCTGC-3’

Hspb1 Forward 5’-GACATGAGCAGTCGGATTGA-3’

Reverse 5’-GGATGGGGTGTAGGGGTACT-3’

Cxcl10 Forward 5’-CCAAGTGCTGCCGTCATTTTC-3’

Reverse 5’-GGCTCGCAGGGATGATTTCAA-3’

Cd44 Forward 5’-ACCTTGGCCACCACTCCTAA-3’

Reverse 5’-GCAGTAGGCTGAAGGGTTGT-3’

Osmr Forward 5’-GTGAAGGACCCAAAGCATGT-3’

Reverse 5’-GCCTAATACCTGGTGCGTGT-3’

Cp Forward 5’-TGTGATGGGAATGGGCAATGA-3’

Reverse 5’-AGTGTATAGAGGATGTTCCAGGTCA-3’

Serpina3n Forward 5’-CCTGGAGGATGTCCTTTCAA-3’

Reverse 5’-TTATCAGGAAAGGCCGATTG-3’

Aspg Forward 5’-GCTGCTGGCCATTTACACTG-3’

Reverse 5’-GTGGGCCTGTGCATACTCTT-3’

Vim Forward 5’-AGACCAGAGATGGACAGGTGA-3’

Reverse 5’-TTGCGCTCCTGAAAAACTGC-3’

Gfap Forward 5’-AGAAAGGTTGAATCGCTGGA-3’

Reverse 5’-CGGCGATAGTCGTTAGCTTC-3’

H2-T23 Forward 5’-GGACCGCGAATGACATAGC-3’

Reverse 5’-GCACCTCAGGGTGACTTCAT-3’

Serping1 Forward 5’-ACAGCCCCCTCTGAATTCTT-3’

Reverse 5’-GGATGCTCTCCAAGTTGCTC-3’

H2-D1 Forward 5’-TCCGAGATTGTAAAGCGTGAAGA-3’

Reverse 5’-ACAGGGCAGTGCAGGGATAG-3’

Ggta1 Forward 5’-GTGAACAGCATGAGGGGTTT-3’

Reverse 5’-GTTTTGTTGCCTCTGGGTGT-3’

Ligp1 Forward 5’-GGGGCAATAGCTCATTGGTA-3’

Reverse 5’-ACCTCGAAGACATCCCCTTT-3’

Gbp2 Forward 5’-GGGGTCACTGTCTGACCACT-3’

Reverse 5’-GGGAAACCTGGGATGAGATT-3’

Fbln5 Forward 5’-CTTCAGATGCAAGCAACAA-3’

Reverse 5’-AGGCAGTGTCAGAGGCCTTA-3’

Ugt1a1 Forward 5’-CCTATGGGTCACTTGCCACT-3’

Reverse 5’-AAAACCATGTTGGGCATGAT-3’

Fkbp5 Forward 5’-TATGCTTATGGCTCGGCTGG-3’

Reverse 5’-CAGCCTTCCAGGTGGACTTT-3’

Psmb8 Forward 5’-CAGTCCTGAAGAGGCCTACG-3’

Reverse 5’-CACTTTCACCCAACCGTCTT-3’

Srgn Forward 5’- GCAAGGTTATCCTGCTCGGA-3’

Reverse 5’-TGGGAGGGCCGATGTTATTG-3’

Amigo2 Forward 5’-GAGGCGACCATAATGTCGTT-3’

Reverse 5’-GCATCCAACAGTCCGATTCT-3’

Clcf1 Forward 5’-CTTCAATCCTCCTCGACTGG-3’

Reverse 5’-TACGTCGGAGTTCAGCTGTG-3’

Tgm1 Forward 5’-CTGTTGGTCCCGTCCCAAA-3’

Reverse 5’-GGACCTTCCATTGTGCCTGG-3’

Ptx3 Forward 5’-AACAAGCTCTGTTGCCCATT-3’

Reverse 5’-TCCCAAATGGAACATTGGAT-3’

S100a Forward 5’-CCTCTGGCTGTGGACAAAAT-3’

Reverse 5’-CTGCTCACAAGAAGCAGTGG-3’

Sphk1 Forward 5’-GATGCATGAGGTGGTGAATG-3’

Reverse 5’-TGCTCGTACCCAGCATAGTG-3’

Cd109 Forward 5’-CACAGTCGGGAGCCCTAAAG-3’

Reverse 5’-GCAGCGATTTCGATGTCCAC-3’

Ptgs2 Forward 5’-GCTGTACAAGCAGTGGCAAA-3’

Reverse 5’-CCCCAAAGATAGCATCTGGA-3’

Emp1 Forward 5’-GAGACACTGGCCAGAAAAGC-3’

Reverse 5’-TAAAAGGCAAGGGAATGCAC-3’

Slc10a6 Forward 5’-GCTTCGGTGGTATGATGCTT-3’

Reverse 5’-CCACAGGCTTTTCTGGTGAT-3’

Tm4sf1 Forward 5’-GCCCAAGCATATTGTGGAGT-3’

Reverse 5’-AGGGTAGGATGTGGCACAAG-3’

B3gnts Forward 5’-CGTGGGGCAATGAGAACTAT-3’

Reverse 5’-CCCAGCTGAACTGAAGAAGG-3’

Cd14 Forward 5’-GGACTGATCTCAGCCCTCTG-3’

Reverse 5’-GCTTCAGCCCAGTGAAAGAC-3’

Cxcl9 Forward 5’-TCCTTTTGGGCATCATCTTCC-3’

Reverse 5’-TTTGTAGTGGATCGTGCCTCG-3’

IL-6 Forward 5’-TAGTCCTTCCTACCCCAATTTCC-3’

Reverse 5’-TTGGTCCTTAGCCACTCCTTC-3’

IL-1β Forward 5’-GCAACTGTTCCTGAACTCAACT-3’

Reverse 5’-ATCTTTTGGGGTCCGTCAACT-3’

Ccl2 Forward 5’-TTAAAAACCTGGATCGGAACCAA-3’

Reverse 5’-GCATTAGCTTCAGATTTACGGGT-3’

Ki 67 Forward 5’-ATCATTGACCGCTCCTTTAGGT-3’

Reverse 5’-GCTCGCCTTGATGGTTCCT-3’

IL-22R Forward 5’- ATGAAGACACTACTGACCATCCT-3’

Reverse 5’- CAGCCACTTTCTCTCTCCGT-3’

**Human:**

GAPDH Forward 5’-GGAGCGAGATCCCTCCAAAAT-3’

Reverse 5’-GGCTGTTGTCATACTTCTCATGG-3’

Cxcl10 Forward 5’-GTGGCATTCAAGGAGTACCTC-3’

Reverse 5’-TGATGGCCTTCGATTCTGGATT-3’

Ccl2 Forward 5’-CAGCCAGATGCAATCAATGCC-3’

Reverse 5’-TGGAATCCTGAACCCACTTCT-3’

Bcl2 Forward 5’-GGTGGGGTCATGTGTGTGG-3’

Reverse 5’-CGGTTCAGGTACTCAGTCATCC-3’
